# Non-Invasive Hemodynamic Monitoring for Hemodynamic Management in Perioperative Medicine

**DOI:** 10.3389/fmed.2017.00209

**Published:** 2017-11-23

**Authors:** Julia Y. Nicklas, Bernd Saugel

**Affiliations:** ^1^Department of Anesthesiology, Center of Anesthesiology and Intensive Care Medicine, University Medical Center Hamburg-Eppendorf, Hamburg, Germany

**Keywords:** cardiovascular monitoring, blood pressure, advanced hemodynamic variables, continuous cardiac output, pulse contour analysis

## Background

In perioperative medicine, hemodynamic management aims at an optimization of perfusion pressure and oxygen delivery in order to maintain or restore adequate cellular metabolism (1). To optimize cardiopulmonary function, hemodynamic management triggers the administration of fluids and vasoactive agents according to predefined target values of hemodynamic variables. This is often referred to as “goal-directed therapy” (GDT). Although the general and vague term GDT comprises various (in part very different) hemodynamic treatment strategies (2), GDT has been shown to improve patient outcome, especially in high-risk patients undergoing major surgery (3–11). Besides basic hemodynamic variables (blood pressure and heart rate), GDT treatment algorithms usually include advanced hemodynamic variables such as pressure- or volume-based cardiac preload variables (central venous pressure, pulmonary capillary wedge pressure, global end-diastolic volume), dynamic cardiac preload variables (pulse pressure variation, stroke volume variation), and blood flow variables (stroke volume, cardiac output). A variety of invasive, less-invasive, and non-invasive hemodynamic monitoring technologies are nowadays available to assess hemodynamic variables in the operating room or the intensive care unit. In this opinion paper, we will discuss how innovative non-invasive hemodynamic monitoring might be used for hemodynamic management in perioperative medicine.

## Hemodynamic Monitoring Technologies Used for GDT

Until recently, the measurement of advanced hemodynamic variables used in GDT protocols required invasive hemodynamic monitoring such as invasive pulse contour analysis (arterial catheter), transpulmonary thermodilution (dedicated arterial catheter and central venous catheter), or pulmonary artery thermodilution (pulmonary artery catheter). However, during the last decades, the use of the pulmonary artery catheter in perioperative medicine and critical care is declining (12, 13) and the routine use of the pulmonary artery catheter is not recommended for surgical patients undergoing non-cardiac surgery (14). Advanced hemodynamic monitoring using the transpulmonary thermodilution technique, often called a less-invasive alternative to the pulmonary artery catheter, is also used only in a minority of patients in the perioperative period (15). Especially in the UK, the esophageal doppler is used to assess blood flow for perioperative GDT (3). Many recent studies on perioperative GDT used un-calibrated invasive pulse contour analysis (arterial catheter) to assess blood pressure, dynamic cardiac preload parameters, or cardiac output (3, 4, 16–18).

In the recent years, different completely non-invasive hemodynamic monitoring technologies were proposed (19). Measurement principles of these innovative hemodynamic monitoring technologies are, among others, bioimpedance and bioreactance, pulse wave transit time, partial carbon dioxide rebreathing, and non-invasive pulse contour analysis (19–27). It is beyond the scope of this article to discuss in detail the underlying measurement principles. In general, the main advantage of these new technologies is that they allow the estimation of cardiac output and other advanced hemodynamic variables without the need for arterial or central venous cannulation. In addition, using these technologies in clinical practice is relatively easy and does not require extensive training. On the other hand, all of the available technologies still have technical limitations with regard to their clinical applicability (19). Furthermore, the numerous validation studies comparing these innovative measurement technologies with established reference methods revealed contradicting results (19, 28–31).

In the following, we want to describe how these innovative technologies can be used for hemodynamic management in perioperative medicine.

## Non-Invasive Hemodynamic Monitoring for Perioperative Hemodynamic Management—Available Data

There are still only a few studies available that investigated the feasibility and usefulness of perioperative GDT based on completely non-invasive hemodynamic monitoring technologies.

In a prospective randomized controlled trial, Benes et al. (32) evaluated the impact of continuous non-invasive blood pressure monitoring using the volume clamp method (finger cuff) on blood pressure stability in patients undergoing thyroid gland surgery in an upright position (“beach chair position”). Patients were randomized to the study group (continuous blood pressure monitoring) or to the control group (intermittent blood pressure monitoring with oscillometric upper arm cuff). Continuous non-invasive blood pressure monitoring significantly decreased the time spent in intraoperative hypotension defined as blood pressure −20% below the individual patient’s target blood pressure (14 vs. 34%; *p* = 0.003). However, the study was too small to adequately evaluate whether this reduction of time spent in hypotension translates into an improvement in postoperative patient outcome.

Fellahi and colleagues (33) evaluated the impact of intraoperative GDT based on stroke volume variation and cardiac index assessed with an endotracheal bioimpedance cardiac output monitor on postoperative outcome after coronary artery bypass surgery in a prospective, controlled, parallel-arm trial. In patients in the study group, the proportion of patients receiving fluid loading and dobutamine was higher compared with the control group. Although the primary endpoint (time to hospital discharge) was not different between the groups, the time to extubation was statistically significantly shorter in the GDT intervention group.

In a similar setting, Leclercq et al. (34) evaluated the feasibility and clinical utility of an endotracheal bioimpedance cardiac output monitoring to optimize intraoperative hemodynamics and improve short-term outcome in patients undergoing off-pump coronary artery bypass grafting surgery. The authors compared 20 patients in whom hemodynamics were monitored with the bioimpedance technology with a historic control of 42 patients. The primary endpoint, the rate of postoperative intensive care unit admission, occurred significantly less often in the bioimpedance group than in the control group (55 vs. 90%; *p* = 0.008). In addition, the time to extubation, the length of stay in the intensive care unit, and the lactate level 6 h after surgery were significantly lower in the bioimpedance group. The authors thus concluded that the systematic use of endotracheal bioimpedance cardiac output monitoring is associated with a reduction in the rate of intensive care unit admission and an improvement in immediate postoperative outcome in patients undergoing off-pump coronary artery bypass grafting surgery.

Broch et al. (35) investigated the feasibility and clinical impact (postoperative complications up to 28 days and length of hospital stay) of GDT based on non-invasive pulse contour analysis (volume clamp method) in patients undergoing elective major abdominal surgery. In their randomized controlled trial, patients in the study group who were treated according to an algorithm based on non-invasively assessed cardiac index and pulse pressure variation were compared with patients in the control group (“standard of care”). The total number of complications was lower in the study group compared with the control group without reaching statistical significance (94 vs. 132; *p* = 0.22). There was also no clinically relevant or statistically significant difference in hospital length of stay or mortality. Thus, the authors conclude that this study demonstrates the general feasibility of a non-invasive GDT approach for hemodynamic optimization in major abdominal surgery. However, following this specific GDT protocol did not improve outcome.

The pleth variability index (i.e., the variability in the pulse oximeter plethysmogram) can be used as a non-invasive dynamic cardiac preload parameter. Forget et al. (36) randomized 82 major abdominal surgery patients into two groups to compare intraoperative fluid management guided by the pleth variability index and mean arterial pressure vs. standard fluid management based on mean arterial and central venous pressure. Interestingly, the amount of intraoperatively administered crystalloids and the total volume of fluids infused were significantly lower in the pleth variability index-GDT group. Lactate levels (primary endpoint) were significantly lower in the GDT group compared with the control group during surgery and 48 h after surgery.

In the multicenter (six tertiary hospitals) randomized clinical POEMAS trial (37), it was evaluated whether perioperative GDT based on non-invasive bioreactance monitoring decreases the incidence of postoperative complications and hospital length of stay in 142 major abdominal surgery patients requiring intensive care unit admission. The GDT protocol included the administration of fluids and vasoactive agents to target values for mean arterial pressure and cardiac index. In the study group, colloid boluses, red blood cell units, and dobutamine was used more often compared with the control group. The study failed to demonstrate a beneficial impact of GDT on patient outcome in terms of overall complications or length of stay between the intervention group and the control group.

We soon will report the results of a monocenter randomized controlled trial in high-risk patients undergoing major abdominal surgery (https://clinicaltrials.gov: NCT02834377) in which we performed “personalized hemodynamic management” (1) by applying a protocol for intraoperative GDT to target the patients’ personal normal cardiac index values as measured the day before surgery using the non-invasive volume clamp method.

## Non-Invasive Hemodynamic Monitoring for Perioperative Hemodynamic Management—Future Perspectives

As discussed above, to date, there are still limited data on the use of non-invasive hemodynamic monitoring technologies for perioperative GDT.

Nevertheless, in the future, these innovative technologies for continuous non-invasive advanced hemodynamic monitoring might offer a variety of opportunities to improve and expand perioperative GDT strategies.

Non-invasive monitoring technologies might enable hemodynamic management strategies to be applied in different new clinical settings (intermediate and low risk surgery) and in patient groups in which advanced hemodynamic monitoring was so far usually not applied (e.g., in patients without arterial catheter or in patients undergoing surgery in regional peripheral or neuraxial anesthesia).

**Figure 1:**
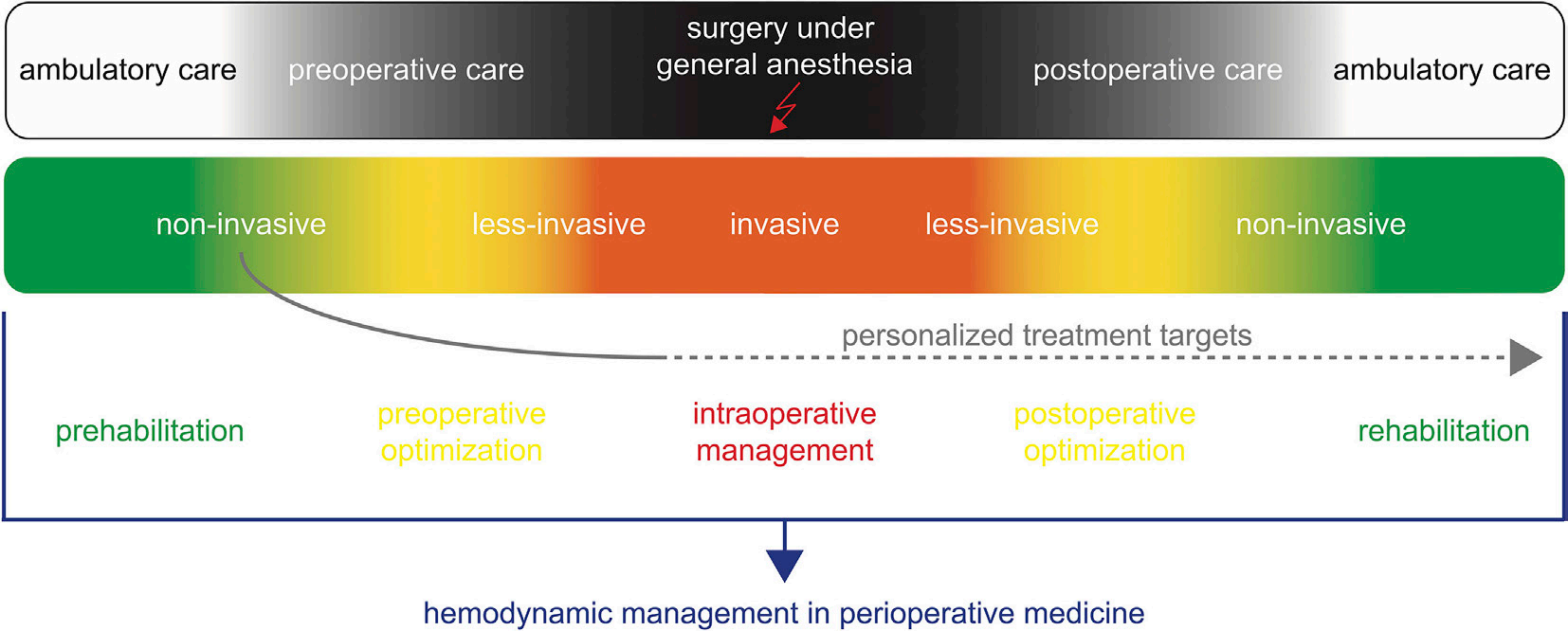
Non-invasive hemodynamic monitoring for hemodynamic management in perioperative medicine. Non-invasive hemodynamic monitoring might be applied for prehabilitation and preoperative optimization during the ambulatory and preoperative care. In addition, it can be used to define personalized targets for the intraoperative hemodynamic management and postoperative optimization.

In addition, with non-invasive monitoring technologies, the patients’ hemodynamic status can be assessed even before induction of anesthesia and after surgery (Figure 1). Non-invasive hemodynamic monitoring might thus be applied for prehabilitation [i.e., optimizing the patient’s hemodynamic status in the weeks before surgery (38)] and preoperative optimization. In addition, values of hemodynamic variables assessed at different time points in the preoperative phase might be used as targets to guide intraoperative hemodynamic management and postoperative optimization (1). The availability of non-invasive technologies for the assessment of advanced hemodynamic variables might thus open a window for perioperative concepts of “personalized hemodynamic management” that aims to optimize cardiovascular dynamics based on the patient’s personal hemodynamic profile (1). Because these innovative technologies enable blood pressure, blood flow, and dynamic cardiac preload variables to be estimated in a completely non-invasive manner even in the preoperative evaluation clinic or on the normal ward, they can be used to determine a patient’s personal normal values of these hemodynamic variables prior to induction of anesthesia and surgery (1). Thus, non-invasive hemodynamic monitoring technologies might help to assess and define personal target values for perioperative GDT in contrast to conventional GDT often using predefined fixed population-based “normal” values as hemodynamic target values (1).

In the future, further technical and digital innovations [e.g., implantable, wireless, or wearable sensors (39, 40)] might further pave the way for GDT based on non-invasive hemodynamic monitoring in perioperative medicine.

## Conclusion

Perioperative hemodynamic management based on the assessment of advanced hemodynamic variables aims at an optimization of cardiovascular dynamics to improve postoperative patient outcome. Until recently, hemodynamic management required invasive hemodynamic monitoring (arterial catheter, central venous catheter, pulmonary artery catheter). Recently, different monitoring technologies that enable advanced hemodynamic variables to be estimated non-invasively became available. In theory, these innovative technologies for continuous non-invasive advanced hemodynamic monitoring might offer a variety of opportunities to improve and expand hemodynamic management strategies and to personalize hemodynamic management (1) in the perioperative phase. However, there are still only a few studies available investigating perioperative GDT based on these innovative technologies with regard to the clinical feasibility and the impact on patient outcome; thus, further research is needed to evaluate and establish non-invasive hemodynamic monitoring for hemodynamic management in perioperative medicine.

## Author Contributions

JYN and BS both contributed substantially to the conception of the work and drafted the manuscript. The authors agree to be accountable for all aspects of the work and ensure that questions related to the accuracy or integrity of any part of the work were appropriately investigated. JYN and BS approved the final version of the manuscript to be published.

## Conflict of Interest Statement

JYN received institutional research grants, unrestricted research grants, and refunds of travel expenses from Tensys Medical Inc. (San Diego, CA, USA). JYN received refunds of travel expenses from CNSystems Medizintechnik AG (Graz, Austria). BS collaborates with Pulsion Medical Systems SE (Feldkirchen, Germany) as member of the medical advisory board and received honoraria for giving lectures and refunds of travel expenses from Pulsion Medical Systems SE. BS received institutional research grants, unrestricted research grants, and refunds of travel expenses from Tensys Medical Inc. (San Diego, CA, USA). BS received honoraria for giving lectures and refunds of travel expenses from CNSystems Medizintechnik AG (Graz, Austria). BS received research support from Edwards Lifesciences (Irvine, CA, USA). The reviewer AS and handling Editor declared their shared affiliation.
